# Superior short-term outcomes of FNS in combination with a cannulated screw in treating femoral neck fractures

**DOI:** 10.1186/s12891-023-06959-w

**Published:** 2023-10-18

**Authors:** Min Su, Zexing He, Nianlai Huang, Xiaocong Lin, Kaibin Fang, Zhangsheng Dai

**Affiliations:** Department of Orthopaedic Surgery, The Second Affiliated Hospital of Fujian Medical University, Quanzhou, Fujian China

**Keywords:** Femoral neck fracture, Femoral neck system, Cannulated screw, Tip-apex distance

## Abstract

**Background:**

This study aimed to evaluate the clinical efficacy of the femoral neck system alone or in combination with a cannulated screw compared with other internal fixation methods for treating femoral neck fractures. We further investigated the predictive effects of tip-apex distance (TAD) on clinical efficacy.

**Methods:**

Data from 129 young adults with femoral neck fractures followed up at The Second Affiliated Hospital of Fujian Medical University between January 2016 and June 2022 were retrospectively collected. The patients were categorized into four groups based on the different internal fixation methods. Analysis and comparisons of the four group were performed according to age, ASA score, operation time, blood loss, fracture classification, fracture healing time, Harris score, TAD value, presence of complications (osteonecrosis of the femoral head, screw failure, and femoral neck shortening), and changes in the neck-shaft angle.

**Results:**

All 129 patients were followed up for at least one year. The group who received treatment with the femoral neck system combined with a cannulated screw exhibited the shortest fracture healing time. Differences were observed in the change of neck-shaft angle among the four groups (P < 0.001), with the smallest change observed in the aforementioned group (0.76 ± 0.54°). The femoral neck shortening was also lower in groups with the femoral neck system or combined with a cannulated screw. At the last follow-up surgery, the combined treatment group achieved the highest HHS score. Subgroup analysis revealed that when the TAD was less than 25 and 49 mm for the femoral neck system and combined groups, respectively, there was less femoral neck shortening, less change in the neck-shaft angle, and a higher HHS score.

**Conclusions:**

The femoral neck system alone or combined with a cannulated screw demonstrated better short-term efficacy in the treatment of femoral neck fractures. Furthermore, TAD may serve as a predictive indicator of the potential success of femoral neck fracture treatment.

## Background

Femoral neck fractures frequently occur in orthopedic clinics and are associated with a high incidence of disability and mortality [1, 2]. As the population ages, the number of older patients with this type of injury continues to increase. Arthroplasty is typically preferable to internal fixation for patients aged ≥ 65 years with femoral neck fractures. However, as time continues to pass after artificial hip replacement, the function of the hip joint gradually diminishes. Additionally, patients may require one or more revision surgeries for artificial hip joints owing to infection, aseptic loosening, or other factors; this eventually increases the financial burden and causes limb damage. For younger patients with greater mobility, internal fixation offers the benefits of reduced trauma, preservation of the femoral head, and improved postoperative hip joint activity. Furthermore, if internal fixation fails, second-stage revision becomes easier. Despite these advantages, the choice of internal fixation remains a challenge in the field of traumatic orthopedics [3–5]. Current internal fixation methods include the use of cannulated screws (CS), dynamic hip screws (DHS), and the femoral neck system (FNS). Among these, triple cannulated screws fixation remains the classic surgical method. The triangular distribution constructed in this technique can create a three-dimensional structure with skeleton and bone tissue, reducing stress on the rotation of the femoral head. This method enhances compressive stress between fracture ends during and after the operation, promotes close contact between the fracture ends, and facilitates fracture healing. However, there is no correlation among the three cannulated screws, and the screw position can be easily influenced by subjective and objective factors related to the surgeon. As a result, its ability to resist vertical shear and torsion is poor, potentially leading to the loosening and displacement of the fracture end, femoral head necrosis and nonunion, and femoral neck shortening. Thus, to improve the stability of internal fixation and achieve better resistance to vertical shear stress, some researchers have made improvements to the structure and number of cannulated screws. In a biomechanical study of cadavers, Kuffman et al. [6]demonstrated that the use of quadruple cannulated screws to stabilize femoral neck fractures significantly reduces axial and anterior displacement compared with using three screws. However, Panteli et al. [7]believed that adding a fourth screw did not provide any biomechanical benefits. Dynamic hip screws have both dynamic and static tension band functions, making them effective at maintaining angle stability, promoting the anatomical reduction of fractures, and accelerating fracture healing [8]. However, recent studies have raised concerns regarding the use of dynamic hip screws, owing to the need for larger surgical incision and increased soft tissue dissection, which can negatively affect the blood supply to the femoral head. Consequently, the incidence of avascular necrosis in the femoral head is higher. In contrast, cannulated screws require smaller surgical incisions and minimal invasion. This allows early functional exercise and rehabilitation. However, the reoperation rate is reportedly higher with the use of cannulated screws compared to dynamic hip screws [8–10]. The FNS, a newly developed internal fixator, comprises a power rod with a diameter of 10 mm and an angle of 130°, with a locking plate placed at the proximal end of the femoral neck system. This is complemented by an anti-rotation screw placed in the same sleeve. The anti-rotation screw in the FNS system has a diameter of 6.4 mm, and an angle of 7.5° with the power rod, creating a screw-in-screw structure. Additionally, one or two 5 mm locking screws were inserted at the distal end of the femoral neck system. Biomechanical tests and finite element analysis have indicated that FNS combines the benefits of minimally invasive cannulated screws while retaining a greater femoral blood supply, as well as the advantages of DHS in terms of angle stability and sliding compression [11, 12]. However, few studies have explored the efficacy of the FNS or FNS combined with a cannulated screw (FNS + CS) for the treatment of femoral neck fractures.

Thus, this study aimed to analyze the clinical effectiveness of the FNS or its combination with a cannulated screw, as well as other internal fixation methods, in the treatment of femoral neck fractures. Additionally, this study aimed to assess the predictive efficacy of the tip-apex distance (TAD) on clinical outcomes. The findings of this study provide evidence-based support for future clinical practice.

## Materials and methods

### Patients

**Fig. 1:**
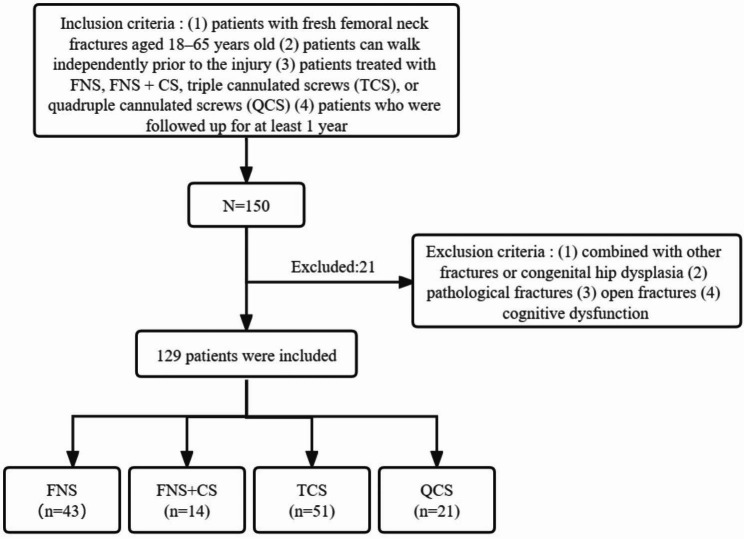
Flow chart of the case selection process

In this retrospective study, we collected data from 129 patients with femoral neck fractures who underwent treatment at our hospital between January 2016 and June 2022 (Fig. 1). The patients were followed up for at least 1 year. The study protocol was performed in compliance with the Helsinki Declaration and approved by the ethics committee of The Second Affiliated Hospital of Fujian Medical University (study no. IRB_2021.213), and all patients provided informed consent prior to participation.

### Inclusion criteria and exclusion criteria

The inclusion criteria were as follows: (1) patients with fresh femoral neck fractures aged 18–65 years old, (2) patients can walk independently prior to the injury, (3) patients treated with FNS, FNS + CS, triple cannulated screws (TCS), or quadruple cannulated screws (QCS), (4) patients who were followed up for at least 1 year.

The exclusion criteria were as follows: (1) combined with other fractures or congenital hip dysplasia, (2) pathological fractures, (3) open fractures, (4) cognitive dysfunction.

### The operation process

Patients were administered intravenous antibiotics 30 min before surgery. After successful anesthesia, the patient was placed in the supine position, and the surgical field was disinfected with a 2.5% tincture of iodine and 75% alcohol. Sterile towels were then applied. For the surgical procedure, the patient was placed on an orthopedic traction bed, and the lower limbs were abducted and internally rotated. Fluoroscopy was used to aid in reducing the fractures, and all surgeries were performed by experienced doctors working cooperatively.

### FNS group

During the surgical procedure, the following steps were taken: (1) Under fluoroscopy, a 2.5 mm Kirschner wire was placed in front of the femoral neck to aid in fracture reduction; (2) A 2.0 mm Kirschner wire was inserted for temporary fixation of the femoral neck fracture; (3) A lateral incision of approximately 4 cm was made on the affected hip, and the skin was cut, followed by layer-by-layer dissection of the subcutaneous tissue, deep fascia, and exposure of the lateral end of the femur; (4) Under fluoroscopy, the FNS plate was placed on the lateral side of the femur, and a compression screw with a 5.0 mm diameter was inserted into the trochanter. In addition, a dynamic rod with a 10 mm diameter and an anti-rotation screw with a 6.4 mm diameter were placed within the femoral neck; (5) The fracture reduction was assessed again using fluoroscopy; (6) Following strict hemostasis, the wound was sutured and bandaged.

### FNS + CS group

Steps 1–4 were the same as those for the FNS group; however, surgery differed thereafter: 5) A Kirschner wire was placed parallel to the dynamic rod above the femoral neck and positioned as close to the upper edge of the femoral neck as possible, with the lateral position located at the center of the femoral neck. Drilling was performed along the guide needle, depth was measured, and a suitable cannulated screw was placed. 6) Fracture reduction was reexamined to ensure satisfactory results. 7) After strict hemostasis, the wounds were sutured and bandaged.

### TCS group

(1) Three guide pins were percutaneously inserted from the lateral side of the upper femur to obtain temporary stability of the fracture; (2) X-ray was used to confirm that the fracture had been reduced in both the anteroposterior and lateral positions of the hip joint, and that the guide pins were located in the femoral neck and reached a 0.5 cm under the cartilage of the femoral head; (3) The length of the screw was measured, and three small incisions were made. The skin was cut, followed by layer-by-layer dissection of the subcutaneous tissue and deep fascia, reaching the lateral side of the femoral greater trochanter; (4) The screw was drilled and screwed into three cannulated screws with a 7.3 mm diameter respectively; (5) The fracture reduction was satisfactory by X-ray examination again; (6) After strict hemostasis, the wound was sutured and bandaged.

### QCS group

Four guide pins (squares) were percutaneously inserted from the lateral side of the upper femur to achieve temporary fracture stability. The diameter of the four cannulated screws was 7.3 mm. If the patient’s femoral neck circumference was small, a cannulated screw with a diameter of 6.5 mm could be used instead. The remaining steps were the same as those in the TCS group.

### Perioperative management

Patients were prophylactically administered first-generation cephalosporins 0.5 h before and after surgery. The decision to use low-molecular-weight heparin sodium to prevent lower extremity venous thrombosis was made based on the VTE score before and immediately after surgery. Following surgery, the affected hip joint was placed in an abduction-neutral position, and local cold therapy was administered to reduce lower limb edema. On the first day post-surgery, a rehabilitation therapist guided patients to start toe and ankle joint movements, perform ankle pump training, complete quadriceps and gluteus maximus isometric contraction training, and assist in hip and knee flexion to prevent pulmonary infection. Patients with osteoporosis were treated with calcium, vitamin D, and calcitonin. After discharge, the patients were routinely administered oral anticoagulants and topical analgesic plasters to alleviate pain. Partial weight-bearing training was performed as the affected limb recovered, with weight-bearing walking allowed for 3–6 months after bone healing. Radiographs were reviewed within 3 days post-surgery, and follow-up radiographs were conducted every month for the first 6 months until healing, followed by every 3 and 6 months after 1 year.

### Clinical outcome indicators

**Fig. 2:**
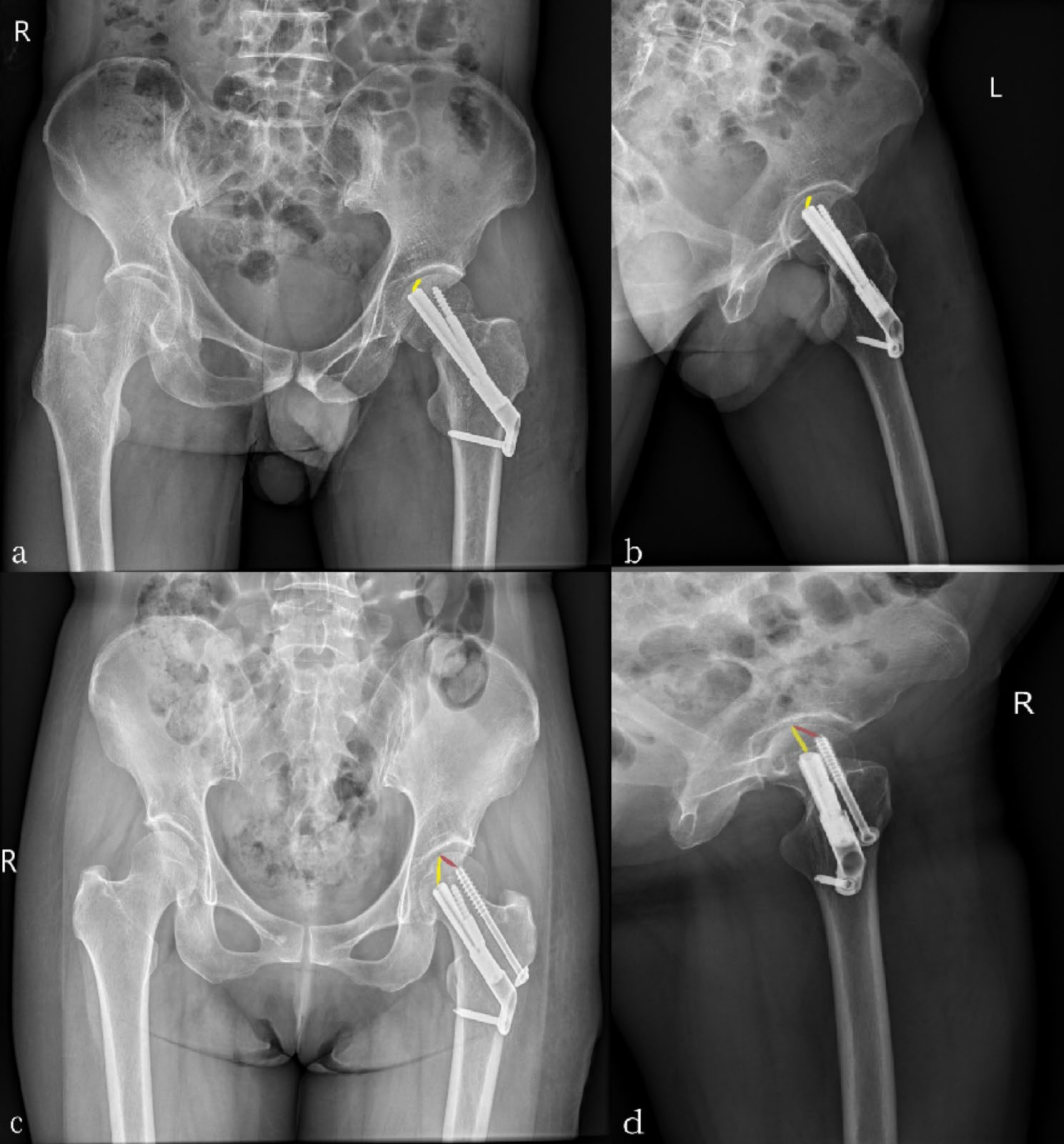
**(a,b)** The measurement method of TAD in the FNS group **(c,d)** The measurement method of TAD in the FNS + CS group

Patients’ medical records were collected from the hospital’s electronic medical record database and imaging system, and factors including age, ASA score, operation time, blood loss, fracture classification (Garden–Pawel classification), fracture healing time, preoperative and postoperative Harris score, and TAD value of the FNS and FNS + CS groups were assessed. Complications (osteonecrosis of the femoral head, screw failure, and femoral neck shortening) and changes in the neck-shaft angle (difference between the neck-shaft angle immediately after surgery and at the last follow-up) were also recorded. Femoral neck shortening was measured using the method described previously [13], with standard pelvic anteroposterior radiographs and a known screw diameter correction magnification. Measurements were performed thrice by the same individual, and the average was obtained. The results were divided into three groups: mild (< 5 mm), moderate (5–10 mm), and severe shortening (> 10 mm). One year post-surgery, hip function was evaluated according to the Harris score standard [14] with a full score of 100 points. Excellent results were defined as scores > 90 points, good as 80–89 points, medium as 70–79 points, and poor as < 70 points. Evaluation of femoral head necrosis was based on the criteria outlined by Slobogean et al. [15], which involves observing the segmental collapse of the femoral head or translucent subchondral areas on radiographs. The TAD was first proposed by Baumgaertner et al. [16] in 1985, and is primarily used for single-screw systems, although some researchers have also applied this technique to double-screw systems. Based on Nuchtern et al. [17], the TAD in the FNS group was determined as the sum of the distance between the tip of the dynamic screw and the intersection of the subchondral bone of the femoral head and the centerline of the femoral neck on anteroposterior and lateral radiographs of the femur immediately after surgery. In the FNS + CS group, the TAD value was determined as the sum of the distances between the dynamic and cannulated screw tips and the intersection of the subchondral bone of the femoral head and the centerline of the femoral neck on anteroposterior and lateral radiographs of the femur immediately post-surgery (Fig. 2).

### Statistical analysis

Continuous variables are reported as mean ± standard deviation (SD), and one-way analysis of variance was used to compare the groups. Categorical variables were analyzed using the chi-squared test or Fisher’s exact probability method. All statistical analyses were performed using SPSS (version 26.0; IBM, Armonk, NY, USA). Statistical significance was set at p < 0.05.

## Results

The patients were divided into four groups based on the internal fixation method: the FNS group (n = 43), FNS + CS group (n = 14), TCS group (n = 51), and QCS group (n = 21). There were no significant differences in age, sex, ASA classification, Garden classification, Pauwels classification, or preoperative Harris score between the groups (Table 1). The operation time of patients treated with FNS and FNS + CS was longer than that of those treated with TCS and QCS (57.33 ± 9.66 min, 61.43 ± 7.95 min, 49.31 ± 9.44 min, 54.76 ± 8.29 min, respectively; p < 0.001). However, no significant differences were observed between the FNS and FNS + CS groups. The intraoperative blood loss was 56.05 ± 18.66 ml in the FNS group, 62.86 ± 17.29 ml in the FNS + CS group, 46.47 ± 22.88 ml in the TCS group, and 48.10 ± 18.61 ml in the QCS group. There was a statistically significant difference in blood loss between the FNS + CS group and the TCS and QCS groups, with more bleeding observed in the former. However, there was no significant difference in blood loss between the FNS and FNS + CS groups, the FNS and QCS groups, or the TCS and QCS groups (Table 2). In the FNS group, screw failure occurred in 1 case (2.3%) and osteonecrosis of the femoral head in 3 cases (7.0%). In the FNS + CS group, screw failure occurred in 0 cases (0.0%) and osteonecrosis of the femoral head occurred in 1 case (7.1%). In the TCS group, 13 patients (25.5%) experienced screw failure, and 10 (19.6%) developed osteonecrosis of the femoral head. In the QCS group, 5 (23.8%) had screw failure, and 4 (19.0%) had osteonecrosis of the femoral head (Table 3). The change in neck-shaft angle was statistically significant among the four groups (p < 0.001), and the smallest change was observed in the FNS + CS group (0.76 ± 0.54°) (Table 3). Of the 43 patients in the FNS group, 32 (74.4%) had no or mild femoral neck shortening, 10 (23.3%) had moderate shortening, and 1 (2.3%) had severe shortening. In the FNS + CS group, 11 (78.6%) patients had no or mild femoral neck shortening, three (21.4%) had moderate shortening, and zero (0.0%) had severe shortening. In the TCS group, 20 patients (39.2%) had no or mild femoral neck shortening, 19 (37.3%) had moderate shortening, and 12 (23.5%) had severe shortening. The QCS group had seven (33.3%) patients with no or mild femoral neck shortening, eight (38.1%) with moderate shortening, and six (28.6%) with severe shortening (Table 3). The postoperative HHS scores were 86.56 ± 2.79, 89.14 ± 2.96, 84.59 ± 3.62, and 82.68 ± 2.89 in the FNS, FNS + CS, TCS group and QCS groups, respectively, at the last follow-up after operation. The FNS + CS group showed the highest HHS score (Table 3).

**Table 1:** Comparison of the general information between the four groups

	FNS	FNS + CS	TCS	QCS	P value
Cases	43	14	51	21	—
Gender(male/female)	26/17	5/9	33/18	14/7	0.241
Age(years)	49.51 ± 10.56	49.21 ± 8.34	45.73 ± 10.46	47.76 ± 8.92	0.303
ASA grading					0.739
I	13	3	19	6	
II	25	8	23	10	
III	5	3	9	5	
Garden type					0.489
I	8	2	14	6	
II	7	2	16	6	
III	15	5	10	5	
IV	13	5	11	4	
Pawels type					0.371
I	7	3	19	6	
II	16	5	18	8	
III	20	6	14	7	
Pre-Op HHS	40.09 ± 4.35	41.29 ± 2.46	41.73 ± 4.19	41.43 ± 3.68	0.241

Abbreviations: ASA American Society of Anesthesiologists, FNS femoral neck system, FNS + CS femoral neck system combined with a cannulated screw, TCS triple cannulated screws, QCS quadruple cannulated screws, Pre-Op HHS preoperative harris hip score

**Table 2:** Comparison of hospitalization and operation between the four groups

	FNS	FNS + CS	TCS	QCS	P value
Operating time (min)	57.33 ± 9.66^ac^	61.43 ± 7.95^a^	49.31 ± 9.44^b^	54.76 ± 8.29^c^	< 0.001
Intraoperative blood loss (ml)	56.05 ± 18.66^ab^	62.86 ± 17.29^a^	46.47 ± 22.88^c^	48.10 ± 18.61^bc^	0.020
Fracture healing(month)	3.88 ± 0.79^a^	3.36 ± 0.93^b^	4.71 ± 0.94^c^	4.38 ± 0.59^c^	< 0.001

Notes: The same letter in the figure indicates no significant difference, while different letters indicate significant difference. Abbreviations: FNS femoral neck system, FNS + CS femoral neck system combined with a cannulated screw, TCS triple cannulated screws, QCS quadruple cannulated screws

**Table 3:** Comparison of postoperative complications and function recovery between the four groups

	FNS	FNS + CS	TCS	QCS	P value
Screw failure	1^a^(2.3%)	0^ab^(0.0%)	13^b^(25.5%)	5^b^(23.8%)	0.001
ONFH	3(7.0%)	1(7.1%)	10(19.6%)	4(19.0%)	0.251
NSA change,°	1.64 ± 0.69^a^	0.76 ± 0.54^b^	3.15 ± 0.84^c^	2.50 ± 0.65^d^	< 0.001
Femoral neck shortening	a	a	b	b	< 0.001
< 5 mm	32(74.4%)	11(78.6%)	20(39.2%)	7(33.3%)	
5-10 mm	10(23.3%)	3(21.4%)	19(37.3%)	8(38.1%)	
> 10 mm	1(2.3%)	0(0.0%)	12(23.5%)	6(28.6%)	
Post-Op HHS	86.56 ± 2.79^a^	89.14 ± 2.96^b^	84.59 ± 3.62^c^	82.68 ± 2.89^d^	< 0.001

Notes: The same letter in the figure indicates no significant difference, while different letters indicate significant difference. Abbreviations: ONFH osteonecrosis of the femoral head, NSA neck shaft angle, Post-Op HHS postoperative Harris hip score, FNS femoral neck system, FNS + CS femoral neck system combined with a cannulated screw, TCS triple cannulated screws, QCS quadruple cannulated screws

TAD values were measured in the FNS and FNS + CS groups, and subgroup analyses were performed using thresholds of 25 and 49 mm, respectively. The results showed that in the FNS group, when the TAD was less than 25 mm, the degree of femoral neck shortening was smaller, the change in neck-shaft angle was smaller, and the postoperative HHS score was higher. Similar results were observed in the FNS + CS group when TAD was less than 49 mm, and the differences were statistically significant (Table 4).

**Table 4:** Comparison of imaging and functional indices between FNS and FNS + CS in different TAD groups

	TAD	NSA change,°	P value	Femoral neck shortening(mm)	P value	Post-Op HHS	P value
FNS	< 25 mm	1.27 ± 0.42	< 0.001	2.03 ± 1.69	< 0.001	87.96 ± 1.73	< 0.001
	> 25 mm	2.11 ± 0.69		4.65 ± 2.59		84.79 ± 2.90	
FNS + CS	< 49 mm	0.48 ± 0.35	0.016	0.85 ± 1.54	0.016	90.63 ± 2.50	0.023
	> 49 mm	1.14 ± 0.54		3.58 ± 2.13		87.17 ± 2.40	

Abbreviations: TAD tip apex distance, NSA neck shaft angle, Post-Op HHS postoperative Harris hip score, FNS femoral neck system, FNS + CS femoral neck system combined with a cannulated screw

## Discussion

**Fig. 3:**
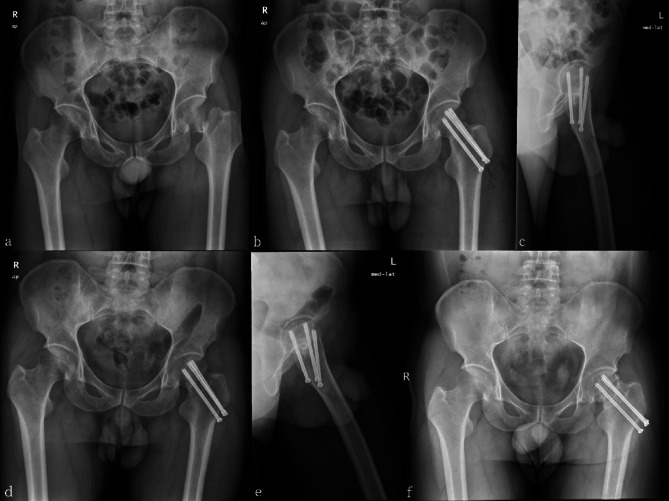
A 33-year-old male patient with left femoral neck fracture was treated with triple cannulated screws(TCS). **(a)** preoperative anteroposterior X-ray image. **(b,c)** postoperative radiographs anteroposterior and lateral images revealing satisfactory reduction of the fracture. **(d,e)** Anteroposterior and lateral X-ray images showing femoral neck shortening and screw withdrawal 1 month after operation. **(f)** Postoperative 16-month follow-up radiographs showing an increased degree of femoral neck shortening

**Fig. 4:**
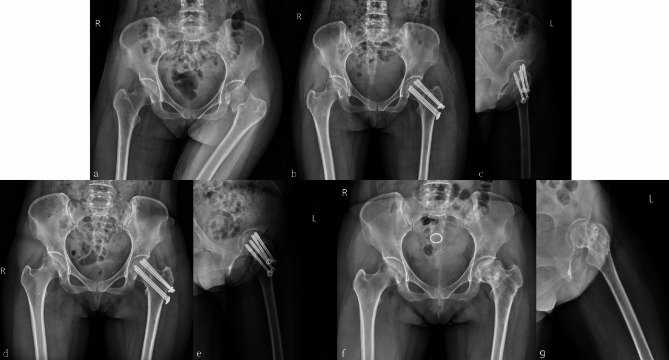
A 37-year-old female patient with left femoral neck fracture was treated with quadruple cannulated screws(QCS). **(a)** Preoperative anteroposterior X-ray image. **(b,c)** Postoperative radiographs anteroposterior and lateral images. **(d,e)** Anteroposterior and lateral X-ray images showing screw withdrawal 1 month after operation. **(f,g)** Postoperative 15-month anteroposterior and lateral radiographs showing that the screw had been removed owing to discomfort and pain in the patient caused by the irritation of the soft tissue by the screw head on the lateral side

Recently, the FNS has emerged as a novel type of internal fixation technique for treating femoral neck fractures. The FNS utilizes an anti-rotation screw and a power rod, which are locked together to ensure anti-rotation. The “nail in nail” design provides an additional layer of anti-rotation, and the 7.5° hangulation of the power rod and anti-rotation screw enhances the overall anti-rotation effect. The use of a fixed plate and power rod also strengthen the stability of the angulation, which effectively prevents reduction loss and facilitates better anti-rotation. Additionally, the FNS was inserted in an impact manner to avoid secondary rotation displacement of the fracture end caused by nail rotation. A previous cadaveric study showed that the femoral neck system had good resistance to varus deformation because of its stable angle [18]. Finite element analysis showed that the femoral neck had the dual effects of overall structural stability and sliding compression, and the biomechanical stability was better than that of the cannulated screws. Furthermore, the biomechanical stability of the FNS is comparable to that of the DHS [11, 19, 20]. Based on previous studies, we concluded that FNS can achieve an effect similar to that of DHS, with strong and stable fixation and preventing postoperative coxa vara [21]. Cannulated screw internal fixation is widely used because of its simple operation, low cost, and strong anti-rotation ability [22]; however, it is associated with clinical problems such as screw failure, femoral neck shortening, osteonecrosis of the femoral head, coxa vara deformity, and high reoperation rates (Figs. 3 and 4). The use of cannulated screws in femoral neck fracture treatment is also common; however, opinions on the number of screws required differ. For comparison, this study included patients who underwent triple and quadruple screw placement.

Effective fixation and reduction of femoral neck fractures are both essential to reduce the incidence of postoperative complications. Failure to achieve stable fixation can lead to displacement, blood supply disorders, and fixation failure, resulting in nonunion and avascular osteonecrosis of the femoral head. This often requires secondary surgery. Femoral head necrosis as a complication after internal fixation has not been applied to clinical effective intervention. Melisik et al. [23] found that bone-sparing joint replacement is a reasonable choice for young patients with high risk factors for osteosynthesis failure. Their results show that ultra-short-stem THA is a feasible treatment option for young patients with femoral neck fractures, with an average clinical survival rate of 94.1%. In the present study, the TCS and QCS groups had higher rates of femoral head osteonecrosis (19.6% and 19.0%, respectively). Previous studies have reported incidence rates of osteonecrosis of the femoral head of 10–30% with any internal fixation method, and our results are similar [24, 25]. Interestingly, although the FNS and FNS + CS groups had a lower rate of osteonecrosis of the femoral head, there was no statistically significant difference in the rate between the four groups. A meta-analysis by Wu et al. [26] also found no significant difference in femoral head necrosis rates between the FNS and TCS groups. This may be because the study included patients aged 18–65 years with a hard femoral neck bone and high bone mineral density, leading to greater fracture damage. The occurrence of femoral head necrosis following femoral neck fractures is related to factors such as the initial fracture displacement, intraoperative reduction quality, internal fixation strength, and postoperative weight-bearing time. Surgeons should strive to achieve perfect reduction during surgery and to use minimally invasive techniques to protect blood supply to the femoral head. As FNS is a new type of internal fixation, it was only introduced to our hospital in 2019, and the follow-up time for some patients may therefore have been shorter than that of the TCS and QCS groups. The blood supply of the femoral head is interrupted after fracture. Although the bone structure heals, reshaping and reconstructing the blood supply remain difficult. As such, evaluation of femoral head necrosis requires follow-up of at least 2 years [27]. Further follow-up is also needed to determine whether the osteonecrosis rate of the femoral head continues to increase in patients receiving FNS and FNS + CS treatment. Additionally, the TCS and QCS groups had higher screw failure rates, with no significant differences between the groups. Previous biomechanics studies [28] have suggested that the addition of a fourth screw does not consistently exert benefits. In the FNS + CS group, the addition of a cannulated screw above the FNS increases screw-screw spacing, reduces stress concentration, and converts high shear forces into favorable compression forces to achieve a more stable fracture end, potentially reducing the occurrence of postoperative internal fixation failure. Furthermore, the change in neck-shaft angle was small in both the FNS and FNS + CS groups, with the FNS + CS group experiencing only a 0.76 ± 0.54° change, which was statistically significant compared to the FNS group. This may be because of the addition of a screw, which can achieve different plane fixations and increased angle stability. Additionally, the force arm at the femoral shaft support increased, ensuring that the distance between the support points was sufficiently large to resist rotation. The cannulated screw can also resist some vertical shear force, and residual shear which is achieved through contact between the locking plate and the lateral femoral cortical bone in the FNS, dispersing stress, effectively ensuring postoperative neck-shaft angle stability and avoiding the occurrence of coxa varus.

**Fig. 5:**
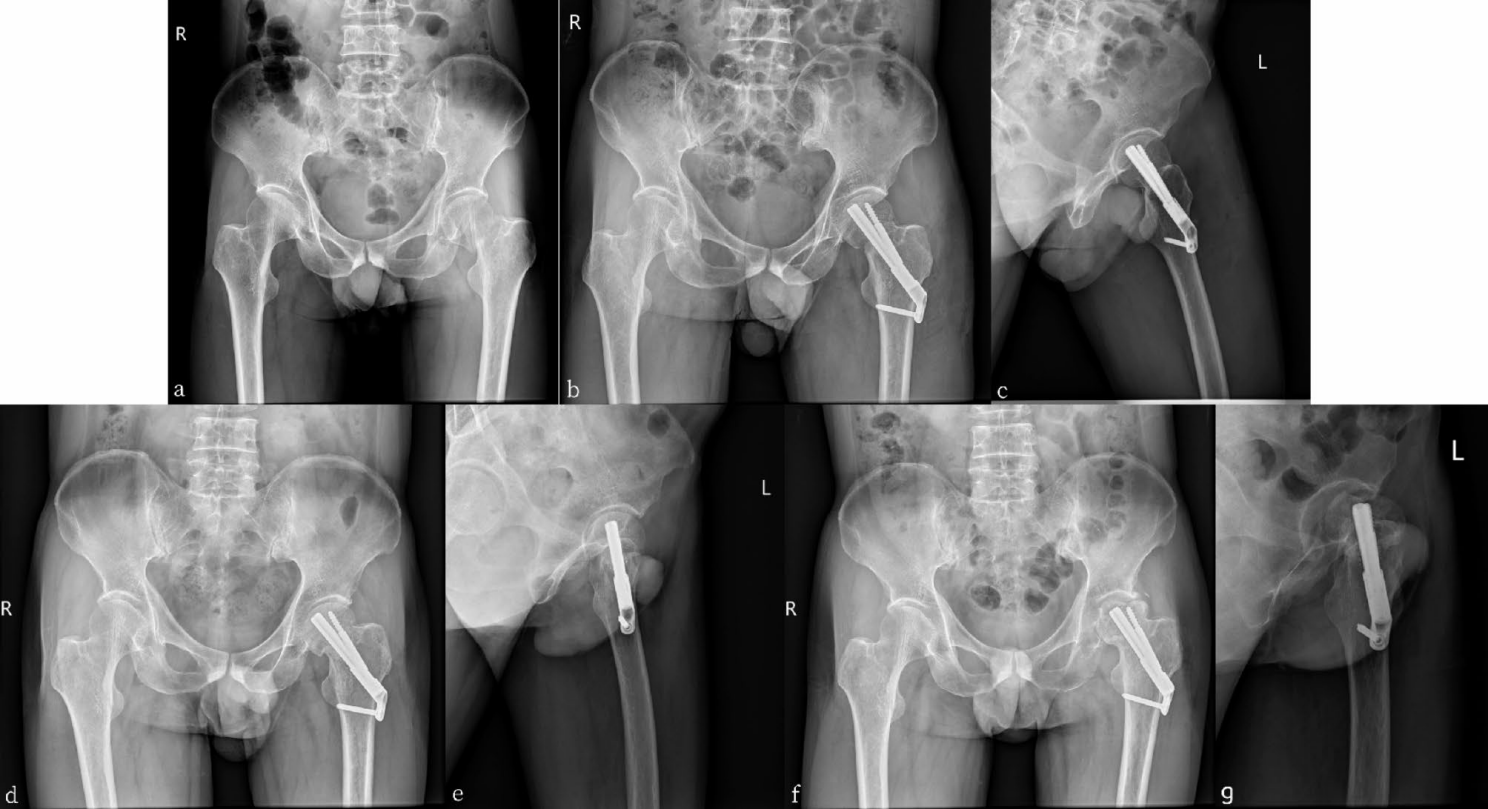
A 52-year-old male patient with left femoral neck fracture was treated with femoral neck systems(FNS). **(a)** Preoperative anteroposterior X-ray image. **(b,c)** Postoperative anteroposterior and lateral radiographs revealing satisfactory reduction of the fracture, and a satisfactory location of the FNS. **(d,e)** Postoperative 2-month anteroposterior and lateral radiographs showing screw sliding in the barrel of the side steel plate. Although the power rod and anti-rotation screw slid, there was no protrusion outside the side plate. **(f,g)** Postoperative 16-month anteroposterior and lateral radiographs showing femoral head necrosis

**Fig. 6:**
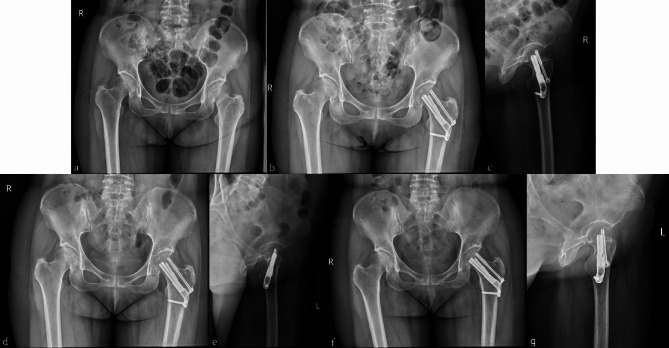
A 53-year-old female patient with left femoral neck fracture was treated with femoral neck systems combined with a cannulated screw (FNS + CS). **(a)** Preoperative anteroposterior X-ray image. **(b,c)** Postoperative anteroposterior and lateral radiographs revealing satisfactory reduction of the fracture and satisfactory location of the FNS + CS. **(d,e)** Anteroposterior and lateral radiographs taken 3 months after the surgery showed that the fracture had healed well. **(f,g)** Anteroposterior and lateral radiographs at 5 months follow-up showed no femoral neck shortening and screw withdrawal, and the neck-shaft angle was maintained well

Femoral neck shortening is a common complication of femoral neck fracture surgery, and its degree is negatively correlated with patient prognosis [29, 30]. The FNS has a sliding compression space of 20 mm, which helps avoid excessive sliding and reduces femoral neck shortening (Fig. 5). In this study, the FNS and FNS + CS groups had significantly lower degrees of femoral neck shortening than the other two groups. The lack of reliable and effective fixation of the cannulated screw on the femoral side can result in nail path retreat once femoral neck shortening occurs, leading to stimulation of the lateral soft tissue of the screw head, and triggering postoperative pain and discomfort (Fig. 4). Harris scores were significantly lower in the TCS and QCS groups, potentially due to this issue. Previous studies have suggested that FNS can accelerate fracture healing, and that the locking mechanism and anti-rotation effect of FNS may provide a more stable structure. Dynamic compression between the fracture ends using FNS may also positively contribute to fracture healing [26, 31]. Similar results were obtained in this study, and we found that the FNS + CS group was superior the FNS group in terms of fracture healing and Harris score (Fig. 6), which may be attributed to the addition of a cannulated screw that provides firmer and more stable fixation. This provides sufficient stability for the fracture site to heal over time, resulting in better functional outcomes. However, the smaller sample size in the FNS + CS group compared to the FNS group may have impacted our results, and larger studies are needed to further confirm these findings. Additionally, both the FNS and FNS + CS groups had longer operation times and more intraoperative bleeding than the cannulated screw group. As the FNS is a newer form of internal fixation, its early clinical practice requires a longer learning curve and the additional placement of a cannulated screw, which needs to be adjusted repeatedly, resulting in prolonged operation time. A larger surgical incision compared to the cannulated screw group also led to increased intraoperative blood loss. However, as surgeons gain more experience with FNS, we believe that the operation time and intraoperative blood loss will ultimately decrease.

TAD is commonly used in the treatment of hip fractures. This technique was first introduced by Baumgaertner et al. [16] for treating intertrochanteric fractures using a dynamic hip screw. They found that when the TAD was < 25 mm, the probability of screw cutout was 0. Although the TAD is primarily used in single-screw systems, it is also used in double-screw systems. Nüchtern et al. [17] found that the upper limit of the TAD for double-screw systems was 49 mm. Khanna et al. [32] further suggested that the TAD of the lag screw should be calculated separately, as the other screw only serves as an anti-rotation screw and does not contribute significantly to fixation. Therefore, we did not measure the anti-rotation screw when calculating TAD for the FNS and FNS + CS groups in our study. While TAD is a traditional method for determining screw position in the femoral head, it has sparked controversy among clinicians. However, because we only observed one screw failure in the FNS group and no failures in the FNS + CS group, we did not investigate the relationship between TAD and the probability of screw cut-out in this study. As such, further research is required to obtain conclusive results. Nevertheless, we found that a TAD < 25 mm in the FNS group and < 49 mm in the FNS + CS group led to better functional scores and radiological results. Therefore, we believe that TAD can be a useful indicator for predicting the effectiveness of FNS with or without a cannulated screw; however, its predictive capacity requires verification based on larger clinical samples.

This study has some limitations. First, the retrospective design may have introduced a selection bias, as the method of internal fixation was selected based on clinical experience. A randomized, multicenter prospective study should be conducted to improve the reliability of our findings. Second, the FNS + CS group had a smaller sample size, and the short clinical application time of the FNS meant that the average follow-up time for both groups was limited. Therefore, we could not determine the incidence of internal fixation failure or femoral head necrosis. Third, during measurement of the neck shaft angle, femoral neck shortening, and TAD, non-standard patient positioning during radiography may have affected the measured values. However, we minimized this potential for error by having a single researcher perform the measurements, whereas the results of three measurements were averaged for each patient.

## Conclusion

In summary, FNS alone or in combination with a cannulated screw can achieve superior short-term outcomes in treating femoral neck fractures compared with the TCS and QCS methods. These outcomes include a shorter fracture healing time and improved maintenance of the neck-shaft angle and femoral neck length, which contribute to improved hip joint function. Furthermore, the FNS + CS group performed better than the FNS group in this respect, and TAD may be a useful predictor of FNS or combined a cannulated screw treatment for femoral neck fractures. Although we found no significant difference in the incidence of femoral head necrosis among the four groups, this study represents only a preliminary investigation, and more extended follow-up studies with larger sample sizes are required. Further, we plan to explore the biomechanical differences between the FNS + CS and FNS using finite element analysis in the future.

## Data Availability

The datasets utilized and analyzed in the present study can be obtained from the corresponding author upon reasonable request.
